# Retinal Structures and Visual Cortex Activity are Impaired Prior to Clinical Vision Loss in Glaucoma

**DOI:** 10.1038/srep31464

**Published:** 2016-08-11

**Authors:** Matthew C. Murphy, Ian P. Conner, Cindy Y. Teng, Jesse D. Lawrence, Zaid Safiullah, Bo Wang, Richard A. Bilonick, Seong-Gi Kim, Gadi Wollstein, Joel S. Schuman, Kevin C. Chan

**Affiliations:** 1 NeuroImaging Laboratory, University of Pittsburgh, Pittsburgh, PA, USA; 2 UPMC Eye Center, Ophthalmology and Visual Science Research Center, Department of Ophthalmology, School of Medicine, University of Pittsburgh, Pittsburgh, PA, USA; 3 Louis J. Fox Center for Vision Restoration, University of Pittsburgh, PA, USA; 4 Department of Bioengineering, Swanson School of Engineering, University of Pittsburgh, PA, USA; 5 Department of Biostatistics, Graduate School of Public Health, University of Pittsburgh, PA, USA; 6 McGowan Institute for Regenerative Medicine, University of Pittsburgh, PA, USA; 7 Center for Neuroscience Imaging Research, Institute for Basic Science, Suwon, Korea; 8 Department of Biomedical Engineering, Sungkyunkwan University, Suwon, Korea; 9 Clinical and Translational Science Institute, University of Pittsburgh, Pittsburgh, PA, USA; 10 Center for the Neural Basis of Cognition, University of Pittsburgh and Carnegie Mellon University, Pittsburgh, PA, USA

## Abstract

Glaucoma is the second leading cause of blindness worldwide and its pathogenesis remains unclear. In this study, we measured the structure, metabolism and function of the visual system by optical coherence tomography and multi-modal magnetic resonance imaging in healthy subjects and glaucoma patients with different degrees of vision loss. We found that inner retinal layer thinning, optic nerve cupping and reduced visual cortex activity occurred before patients showed visual field impairment. The primary visual cortex also exhibited more severe functional deficits than higher-order visual brain areas in glaucoma. Within the visual cortex, choline metabolism was perturbed along with increasing disease severity in the eye, optic radiation and visual field. In summary, this study showed evidence that glaucoma deterioration is already present in the eye and the brain before substantial vision loss can be detected clinically using current testing methods. In addition, cortical cholinergic abnormalities are involved during trans-neuronal degeneration and can be detected non-invasively in glaucoma. The current results can be of impact for identifying early glaucoma mechanisms, detecting and monitoring pathophysiological events and eye-brain-behavior relationships, and guiding vision preservation strategies in the visual system, which may help reduce the burden of this irreversible but preventable neurodegenerative disease.

Glaucoma is an irreversible neurodegenerative disease of the visual system and is the second leading cause of blindness in the world^1^. The prevalence of this age-related disease is expected to increase substantially in coming years because of the aging population^2^. Currently, the only clinically proven glaucoma intervention aims to lower intraocular pressure (IOP) in afflicted individuals. However, glaucoma may continue to progress in some patients even after lowering IOP to normal levels, which indicates that other key factors may be contributing to the disease^3^. When partial vision loss or blindness results from glaucoma, the cost of treating the disease increases by at least 46%^4^.

Although glaucoma is commonly considered an eye-only disease, increasing evidence suggests the involvement of the brain’s visual system in glaucoma^5^,^6^,^7^,^8^,^9^,^10^,^11^,^12^. As vision loss from glaucoma is permanent and cannot be reversed, research in the realm of early diagnosis and monitoring for signs of progression is continuously advanced through the development of techniques for measuring the anterior visual pathways within the eye. For example, our recent study using optical coherence tomography (OCT) showed that substantial structural loss in the retinal nerve fiber layer (RNFL) of the eye appears to be necessary for vision loss to be detected^13^. However, the cause and pathogenesis of glaucoma in both the eye and the brain are still largely undetermined. It further remains unclear whether early changes in other structures of the eye and in the brain may be present before clinical signs are observed. These basic gaps in knowledge must be filled before early intervention and targeted treatment of both the eye and the brain can be exploited for vision preservation or restoration. Herein we aim to understand the mechanisms of glaucoma in the visual system by investigating its structural, metabolic and functional changes in patients with different degrees of vision loss using non-invasive and multi-modal magnetic resonance imaging (MRI) techniques in a 3-Tesla human scanner. We also characterized the eye-brain-behavior relationships by comparing MRI measures with glaucoma disease severity assessed clinically via spectral-domain OCT and Humphrey visual field tests. Our central hypothesis is that visual impairments in glaucoma involve early deterioration in both the eye and the brain that can be detected and monitored by non-invasive multi-modal imaging techniques.

## Results and Discussion

### Study participants with varying degrees of glaucoma severity were matched on age and sex

Thirteen early glaucoma [age = 62.4 ± 2.1 years (mean ± standard error of the mean); 53.9% male] and 13 advanced glaucoma patients (age = 63.6 ± .2.3 years; 38.5% male) were recruited in this study through the patient population at the University of Pittsburgh Medical Center (UPMC) Eye Center and the Clinical Trials Registry at the University of Pittsburgh. All recruited patients were diagnosed with chronic glaucoma. Nine healthy subjects (age = 61.3 ± 3.1 years; 33% male) were also recruited as controls with no statistically significant differences in mean age and in sex composition between the three groups using an ANOVA (p = 0.61) and a χ^2^ test (p = 0.58), respectively. The mean RNFL thickness for our healthy, early glaucoma and advanced glaucoma subjects was 88.3 ± 3.2 μm, 77.2 ± 3.3 μm and 63.3 ± 2.2 μm respectively in the worse eye (mean ± standard error of the mean; ANOVA, p < 0.0001); the mean ganglion cell-inner plexiform layer (GCIPL) thickness was 78.2 ± 3.1 μm, 70.5 ± 2.5 μm and 58.7 ± 2.2 μm respectively in the worse eye (mean ± standard error of the mean; ANOVA, p < 0.0001); the average cup-to-disc ratio was 0.44 ± 0.07, 0.69 ± 0.03 and 0.81 ± 0.02 respectively in the worse eye (mean ± standard error of the mean; ANOVA, p < 0.0001); and the visual field mean deviation was −0.76 ± 0.37 dB, −1.89 ± 0.61 dB and −7.51 ± 1.32 dB respectively in the worse eye (mean ± standard error of the mean; ANOVA, p < 0.0001). The intraocular pressure for our healthy, early glaucoma and advanced glaucoma subjects was 14.11 ± 1.05 mmHg, 13.54 ± 0.73 mmHg and 12.00 ± 1.06 mmHg respectively in the left eye (mean ± standard error of the mean; ANOVA, p = 0.29); and 13.78 ± 0.62 mmHg, 12.31 ± 0.77 mmHg and 13.08 ± 0.90 mmHg respectively in the right eye (mean ± standard error of the mean; ANOVA, p = 0.48).

### Loss of functional activation in the visual cortex is associated with decreased inner retinal thickness across subjects with varying glaucoma severity

**Figure 1:**
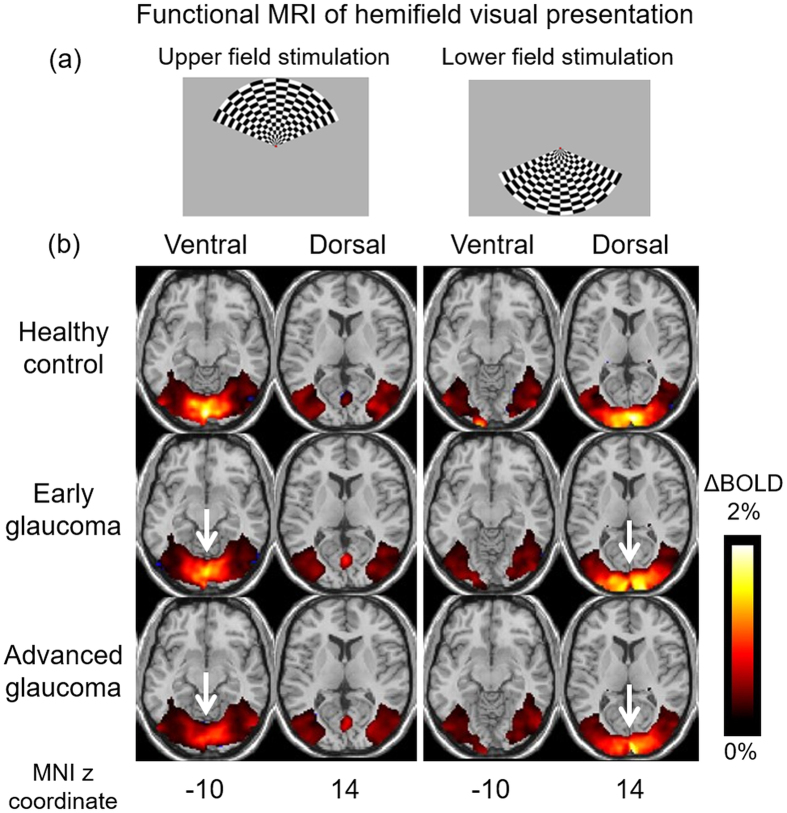
Functional MRI of visual cortex activity in glaucoma and healthy subjects. (**a**) 8 Hz flickering checkerboard patterns were presented to the upper (left column) or lower hemifield (right column) of each subject one eye at a time using the MRI-compatible visual occlusion spectacles (Translucent Technologies, Inc., Toronto, Ontario, Canada) and the e-prime software (Psychology Software Tools, Inc., Sharpsburg, PA, USA). (**b**) Group-wise mean blood-oxygenation-level-dependent (BOLD) brain activation maps of healthy control (top row, n = 9), early glaucoma (middle row, n = 13) and advanced glaucoma subjects (bottom row, n = 13) (Family-wise error corrected p < 0.01). BOLD percent change (ΔBOLD) represents the amount of brain BOLD activity changes during task periods relative to resting periods. Upper visual field stimulation (first 2 columns) predominantly activated the ventral visual cortex, whereas lower visual field stimulation (last 2 columns) predominantly activated the dorsal visual cortex to a generally stronger extent than upper visual field stimulation due to asymmetry in spatial visual field responses^45^. Note the diminishing brain activity with increasing severity in glaucoma especially in the primary visual cortex (arrows). (MNI: Montreal Neurological Institute).

**Figure 2:**
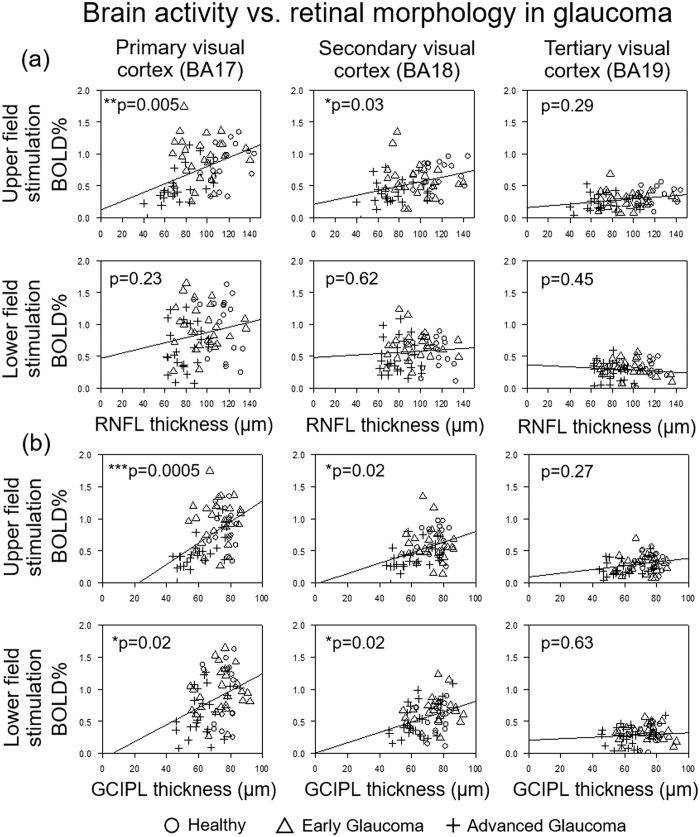
Relationships between brain activity and eye morphology in glaucoma. Relationships between visual cortex activity by BOLD functional MRI (y-axis) and (**a**) peripapillary retinal nerve fiber layer (RNFL) thickness, or (**b**) macular ganglion cell-inner plexiform layer (GCIPL) thickness measured by optical coherence tomography (OCT) (x-axis). Using linear mixed-effects modeling, superior and inferior RNFL and GCIPL thicknesses were in general most strongly associated with BOLD activity in the corresponding hemifields in primary visual cortex [Brodmann Area (BA) 17], less in secondary visual cortex (BA18), and were not significantly associated in tertiary visual cortex (BA19). BOLD responses also appeared to fit tighter with macular GCIPL than peripapillary RNFL throughout the visual cortex. (*p < 0.05, **p < 0.01, ***p < 0.001: estimated slopes are significantly different from 0 by t test).

In order to investigate how glaucoma affects brain function, we evaluated the cortical responses to 8 Hz flickering stimulation patterns in our glaucoma and healthy subjects via a functional MRI experiment using the intrinsic blood-oxygen-level-dependent (BOLD) contrast (Fig. 1). As shown in Fig. 1b, stimulation of either the upper or lower visual hemifield showed decreasing BOLD activity in the retinotopically mapped areas of the visual cortex in patients with increasing glaucoma severity. In particular, the primary visual cortex appeared to exhibit larger disease-driven activity loss compared to the higher-order visual areas. When comparing brain activity to segmented retinal layer thickness in the eye obtained by OCT (Fig. 2), a tighter fit was obtained for the macular GCIPL than the peripapillary retinal nerve fiber layer (RNFL). We speculate that this observation is due to the relatively large proportion of sensory input from the macula to the tested location in the brain as compared to input from the rest of the retina. Whereas the GCIPL thickness reflects structure within the macula, RNFL thickness is related to a more diffuse distribution throughout the eye. In addition, deterioration of both peripapillary RNFL and macular GCIPL in the inner retina appeared more strongly associated with reduced activity in primary visual cortex [Brodmann Area (BA) 17] than higher-order vision-processing centers in secondary (BA18) and tertiary visual cortices (BA19). These results suggest that the primary visual cortex is more severely affected than higher-order cortical regions in glaucoma.

### Retinal thinning, optic nerve cupping and loss of visual cortex activation precede measurable visual field loss

**Figure 3:**
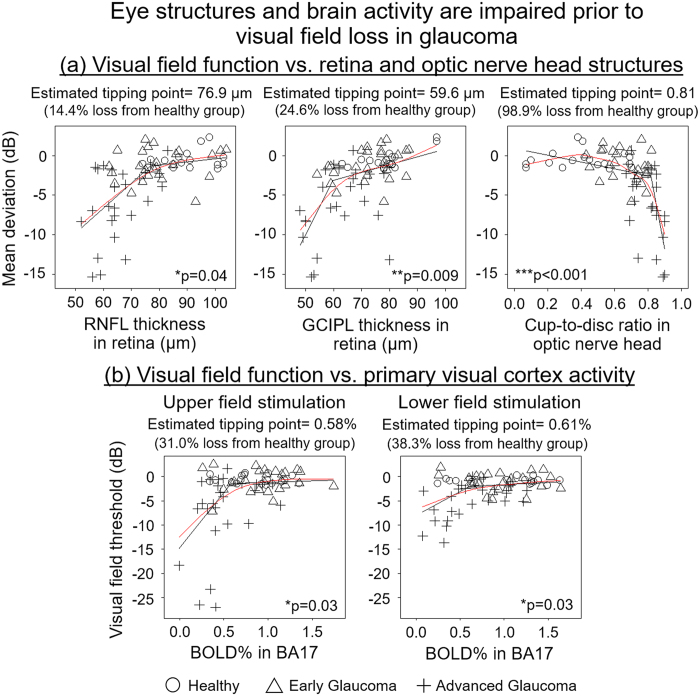
Relationships between visual field impairment and (**a**) eye morphology or (**b**) visual cortex activity in glaucoma. Retinal structure and visual outcome relationships in glaucoma have been described by a broken-stick (segmented) model, in which detectable visual field functional loss emerges after substantial retinal degeneration in the peripapillary retinal nerve fiber layer (RNFL) reaches a tipping point^13^. Here, we compared Humphrey visual field mean deviation with optical coherence tomography-measured peripapillary RNFL thickness, macular ganglion cell-inner plexiform layer (GCIPL) thickness, and optic nerve head cup-to-disc ratio in **(a)**, and confirmed the existence of a tipping point at 76.9 μm for RNFL [95% confidence interval (C.I.) = 67.9 to 86.0 μm], 59.6 μm for GCIPL (95% C.I. = 54.5 to 64.7 μm) and 0.81 for cup-to-disc ratio (95% C.I. = 0.79 to 0.83) among our glaucoma and healthy subjects. Our data in (**b**) also demonstrated a broken-stick model relationship between visual field function and blood-oxygenation-level-dependent (BOLD) brain activity in the primary visual cortex [Brodmann Area (BA) 17] at a tipping point of BOLD = 0.58% for upper visual field stimulation (95% C.I. = 0.34 to 0.82%) and BOLD = 0.61% for lower visual field stimulation (95% C.I. = 0.30 to 0.92%), indicating substantial reduction in brain activity before detectable visual field functional loss. The loss from healthy group at the tipping point in the brackets was calculated from the percentage difference between the estimated tipping point and the average value of the healthy group. Red line represents the spline fit, and the black line represents the broken fit model. (*p < 0.05, **p < 0.01, ***p < 0.001: Davies’ test for statistically significant difference in slope between segments).

To further determine the relationships between eye, brain and visual impairment in glaucoma, we compared the degree of vision loss in visual field assessment with ocular morphology in OCT and visual cortex activity in functional MRI using the broken-stick model (Fig. 3)^13^. Consistent with previous research^13^,^14^, thinning of peripapillary RNFL occurred in our cohort before patients demonstrated measurable visual field impairment on standard automated static perimetry (SAP) testing (Fig. 3a). Furthermore, we looked beyond the RNFL into other ocular structural measures, and a tipping point was detected between visual field function and macular GCIPL thickness and optic nerve head cupping (Fig. 3a), indicating substantial structural loss throughout the inner retina and optic nerve head before functional visual field defects become detectable. Our identified tipping point at the cup-to-disc ratio of 0.8 in the optic nerve head nicely concurred with the threshold that is often used for clinical definition of glaucoma. In addition, we note that the primary visual cortex activity was reduced before patients showed measurable visual field loss (Fig. 3b). The functional form of this relationship indicates that visual field performance remains at a steady level even as cortical activation begins to diminish, whereas visual field loss becomes detectable when reduction in visual cortex activation advances beyond a tipping point. These observations suggest that cortical activation loss, just as with RNFL thinning^13^,^14^, precedes detectable visual field loss. When comparing the percentage loss of eye structures and brain activity at the tipping points relative to the healthy group (Fig. 3b), the peripapillary RNFL appeared to be more susceptible to glaucomatous damages and required less structural loss than the macular GCIPL before visual field loss could be detected. The activity loss in the primary visual cortex at the tipping point appeared more comparable to the structural loss in macular GCIPL than peripapillary RNFL, indicative of a stronger association between primary visual cortex activity and macular GCIPL in contributing to visual field loss. We would like to note that it remains inconclusive to directly infer the onset threshold of glaucoma from the determined tipping points. Instead, it is likely that there are differences in the sensitivity of current measuring methods, such that when OCT and fMRI measurements showed initial structural loss in the eye and initial change in visual cortex activity, the corresponding clinical visual field loss became detectable only after reaching a certain threshold as shown in the current study.

### Glaucoma-driven alterations in brain metabolism are associated with both eye and brain structures and visual function

**Figure 4:**
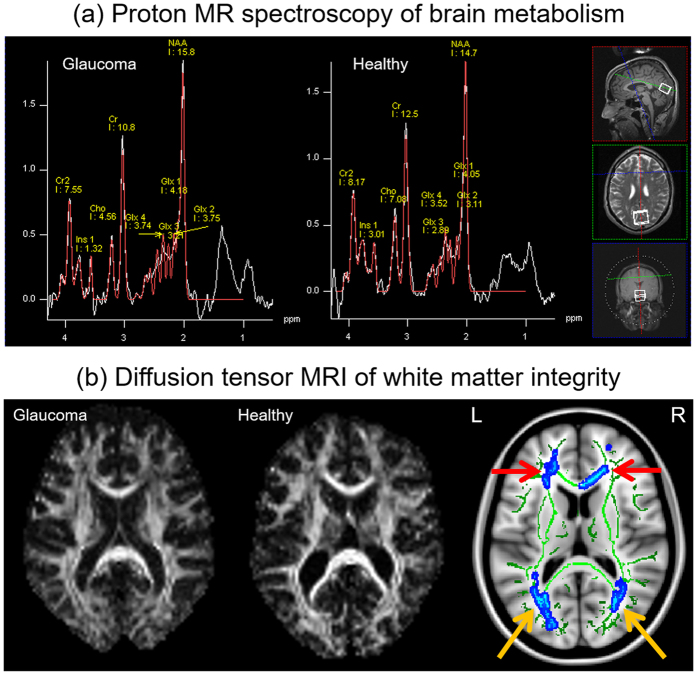
Brain metabolism and brain microstructures in glaucoma. (**a**) Sample proton MR spectrum (white curve) and fitted spectrum (red curve) of the metabolic profiles in the primary visual cortex of a glaucoma patient (left) and a healthy subject (right). Each proton MR spectrum was acquired using a 20 × 25 × 30 mm^3^ voxel centered at the calcarine sulcus bilaterally as shown in the anatomical images in 3 orthogonal planes (white boxes in insets) to cover both upper and lower visual field representations in both hemispheres. The levels of choline (Cho), N-acetyl-aspartate (NAA), glutamate-glutamine complex (Glx) and creatine (Cr) brain metabolite contents were estimated from the spectrum and normalized to the Cr level for quantitative comparisons. (**b**) Sample fractional anisotropy maps of a glaucoma patient (left) and a healthy subject (middle) at the level of the optic radiation, and group comparisons of white matter integrity in the brains of glaucoma patients using tract-based spatial statistics (TBSS) of fractional anisotropy maps (right) in diffusion tensor MRI. Green pixels represent the fractional anisotropy skeletons of major tracts overlaid on the anatomical T1-weighted brain images in grayscale. Blue pixels indicate white matter tract regions that showed significantly lower fractional anisotropy in advanced glaucoma compared to early glaucoma patients (threshold-free cluster enhancement corrected, p < 0.05). The optic radiation (yellow arrows) of advanced glaucoma patients showed significantly lower fractional anisotropy than early glaucoma patients in both hemispheres, indicative of trans-neuronal degeneration of brain microstructures. The white matter in the frontal lobe (red arrows) also exhibited lower fractional anisotropy in more advanced glaucoma patients, supportive of a recent hypothesis of widespread structural brain changes beyond the visual system in glaucoma. No apparent difference in fractional anisotropy was observed between early glaucoma and healthy subjects (data not shown).

**Figure 5:**
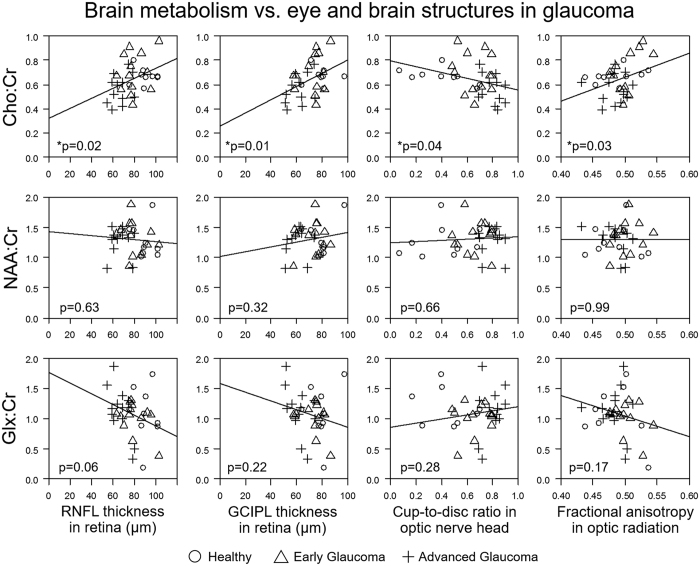
Relationships between brain metabolism and eye and brain structures in glaucoma. Relationships between visual cortex metabolism by proton MR spectroscopy (y-axis) and eye (first 3 columns) and brain structures (last column) by optical coherence tomography (OCT) and diffusion tensor MRI, respectively (x-axis). Positive correlations were found when comparing choline level (using Cho:Cr) with peripapillary retinal nerve fiber layer (RNFL) thickness, macular ganglion cell-inner plexiform layer (GCIPL) thickness and fractional anisotropy in optic radiation, whereas negative correlation was observed when comparing Cho:Cr with optic nerve head cup-to-disc ratio. No apparent correlation was found when comparing N-acetyl-aspartate (using NAA:Cr) or glutamate-glutamine complex (using Glx:Cr) level with OCT or diffusion tensor MRI parameters. Note that OCT measurements were averaged between both eyes and then compared with the proton MR spectroscopy data. (*p < 0.05: estimated slopes are statistically significantly different from 0 by t test) (Cr: creatine).

**Figure 6:**
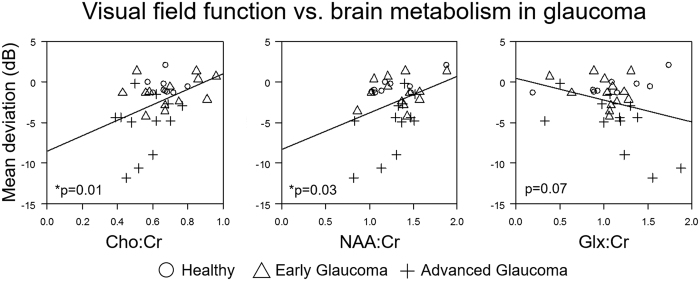
Relationships between Humphrey visual field mean deviation (y-axis) and visual cortex metabolism by proton MR spectroscopy (x-axis). Positive correlations were found when comparing mean deviation with choline and N-acetyl-aspartate levels (using Cho:Cr and NAA:Cr ratios), whereas a negative trend was observed when testing for correlation between mean deviation with glutamate-glutamine complex level (using Glx:Cr ratio). Note that visual field measurements were averaged between both eyes and then compared with the proton MR spectroscopy data. (*p < 0.05: estimated slopes are statistically significantly different from 0 by t test) (Cr: creatine).

In order to understand the possible causes of reduced brain activity in glaucoma, we measured the neurochemical profiles in the visual cortex using proton MR spectroscopy with a voxel-of-interest centered at the calcarine sulcus to cover both upper and lower hemifield regions of the primary visual cortex bilaterally (Fig. 4a). Peak integrals of brain metabolites [N-acetylaspartate (NAA), creatine (Cr), choline (Cho), glutamate/glutamine complex (Glx)] were determined using the syngo MR software (Siemens, Erlangen, Germany). The OCT and visual field parameters were averaged between both eyes and then compared with the brain metabolite peak integrals using the Cr signals as a reference for normalization. We also measured the white matter integrity in the optic radiation using diffusion tensor MRI for comparison with brain metabolism in proton MR spectroscopy. Our diffusion tensor MRI study showed compromised structural integrity in both the optic radiation (Fig. 4b, yellow arrows) and the white matter in the frontal lobe (Fig. 4b, red arrows) of more advanced glaucoma patients, reflective of trans-neuronal degeneration of brain microstructures within^15^,^16^,^17^,^18^,^19^,^20^,^21^ and beyond^22^,^23^,^24^ the visual pathways in glaucoma. Consistent with our preclinical proton MR spectroscopy study in an experimental animal model of chronic glaucoma^25^, our glaucoma patients showed lower levels of Cho in the primary visual cortex with increasing glaucoma severity as assessed by structural measures (i.e., retinal thinning, optic nerve cupping, and compromise in white matter integrity in optic radiation, Fig. 5), and by visual field assessment (Fig. 6). No apparent relationship was found when comparing NAA:Cr or Glx:Cr in proton MR spectroscopy with OCT or diffusion tensor MRI parameters (p > 0.05) (Fig. 5). Nevertheless, significantly lower NAA content and a trend of higher Glx content were observed with increasing visual field functional loss (Fig. 6).

As in our animal model proton MR spectroscopy study^25^, cortical cholinergic abnormalities may be important and sensitive to glaucomatous neurodegeneration. Cholinergic stimulation has been shown to alleviate clinical symptoms and enhance functional brain responses to visual tasks in other neurodegenerative diseases such as Alzheimer’s^26^,^27^,^28^,^29^. Similarly, our results demonstrate that the cholinergic and glutamatergic neurotransmission systems may be involved in the mechanisms of vision loss in the visual cortex of patients with glaucoma. Overall, these results merit more detailed study on the role of cholinergic stimulation^30^,^31^,^32^, such as intervention with choline supplements as a potential targeted treatment to improve visual cortical responses and visual outcomes in glaucoma patients^33^,^34^,^35^,^36^,^37^.

### Limitations and future directions

As this study is an early step in understanding the role of the visual system in the pathogenesis of glaucoma, it has limitations that will be the subject of future investigation. The sample size is limited by the stringent inclusion criteria, which we believe are necessary to ensure that the findings are in fact due to glaucoma. Furthermore, by using cross-sectional data, this study infers the relationships between function, structure and metabolism in the brain with respect to established measures of glaucoma disease severity by comparing these novel imaging measurements with clinical visual field assessments and morphological measurements of the retina and optic nerve head. To measure the progression of these biomarkers in the brain with respect to the disease more accurately, as well as to improve the statistical power, longitudinal measurements in a larger sample over years will be necessary given that glaucoma is a slowly progressing disease. Finally, the underlying mechanisms behind these reported measurements remain to be further elucidated. For example, it is possible that the decreased activation of the visual cortex is due to decreased visual input from the retina, degeneration in the visual cortex, or some combination of these two effects. Mechanistic lines of investigations may be further addressed in longitudinal studies and in animal models of the disease.

## Conclusions

In summary, this study demonstrates structural, metabolic and functional glaucoma-driven changes in both the eye and the brain, as well as the strong relationships between clinical ophthalmic assessments and MRI-based measures of brain integrity in subjects with varying severity of glaucoma. Our results show that glaucomatous degeneration of inner retinal structures is more closely associated with reduced activity in the primary visual cortex than higher-order visual areas. Current data also extend our previously described broken-stick models comparing functional visual field loss in glaucoma with structural loss not only in the peripapillary retinal nerve fiber layer but also in the macular ganglion cell-inner plexiform layers and the optic nerve head. More importantly, a tipping point also exists between visual cortex activity and visual field function, indicating that glaucoma deterioration is already present in the eye and the brain before substantial vision loss can be detected clinically in patients using current testing methods. Metabolically, cortical cholinergic abnormalities may play a role in trans-neuronal degeneration in glaucoma and warrant further investigation on targeting the cholinergic nervous system for glaucoma treatment. The current results can provide important *in vivo* biomarkers for identifying early glaucoma mechanisms, detecting and monitoring pathophysiological events and eye-brain-behavior relationships in glaucoma, and guiding therapeutic strategies to the visual system, which may help reduce the burden of this leading cause of irreversible but preventable visual impairment.

## Methods

### Experimental design

This work describes a cross-sectional observational study in which the structural, metabolic and functional properties in the brain were measured via a multi-modal MRI exam, and modeled against established measures of glaucoma severity using clinical vision assessment and morphology of the retina and optic nerve head in a group of subjects with varying severity of the disease. The institutional review board and ethics committee of the University of Pittsburgh approved this study. This study followed the tenets of the Declaration of Helsinki and was conducted in compliance with the Health Insurance Portability and Accountability Act. Glaucoma patients were recruited in collaboration with the institutional research registry at the UPMC Eye Center, with all diagnoses confirmed either by chart review or direct examination by one of the clinician investigators (IPC or JSS). Subjects were ≥40 years of age, had a visual acuity of 20/60 or better, and a spherical equivalent refractive error between −6.00 and +6.00 D. Subjects were excluded from the study if they had a history of diabetes, any macular pathology, any conditions affecting visual field and retinal thickness other than glaucoma, a history of ocular trauma or surgery other than uncomplicated glaucoma interventions, or cataract extraction. Additionally, subjects were excluded for the use of any medication known to affect the retina. Glaucomatous eyes were classified by characteristic glaucoma damage to the optic nerve and retinal nerve fiber layer with corresponding visual field loss. Glaucoma staging criteria were based on recommendation by the American Glaucoma Society ICD-10 coding guidelines. In brief, early glaucoma consisted of patients with optic nerve damage and visual field changes involving one hemifield but not including the central 10 degrees of fixation, and advanced glaucoma consisted of patients with optic nerve damage and visual field changes involving both hemifields and/or the central 10 degrees of fixation. Exclusion criteria for MRI scans included pregnant or breastfeeding women, presence of implanted metallic or ferromagnetic objects, noticeable anxiety and claustrophobia that would prevent functional neuroimaging, obesity preventing placement in MRI scanner, best-corrected visual acuity in either eye of worse than 20/40, and any ocular or neurological pathology other than glaucoma. Clinical ophthalmic data from a comprehensive eye exam were used for comparisons with brain MRI results. These included spectral-domain OCT of peripapillary retinal nerve fiber layer (RNFL) thickness, macular ganglion cell-inner plexiform layer (GCIPL) thickness and optic nerve head cup-to-disc ratio (Cirrus SD-OCT platform, Zeiss Meditec), as well as Humphrey visual field scores (HVF 24–2 SITA standard protocol, Zeiss Meditec). Only measures meeting standard reliability criteria were used for analysis. The RNFL and GCIPL thicknesses and the cup-to-disc ratio were determined from the device software automatically. During data analysis, parameters derived from the OCT, MRI and visual field data across disease stages were treated as continuous rather than ordinal values, as the intent of this stratification was simply to provide a target for recruitment to ensure a wide variety of glaucoma damage.

### MRI protocol

All MRI experiments were performed on a 3-Tesla Allegra head scanner (Siemens, Erlangen, Germany) at the Neuroscience Imaging Center at the University of Pittsburgh after obtaining informed written consent. Conventional anatomical MRI was first performed covering the whole brain with T1-weighted MRI using a 3D MPRAGE pulse sequence with the following parameters: repetition time (TR) = 1.4 s, echo time (TE) = 2.5 ms, inversion time (TI) = 800 ms, flip angle = 8°, field of view = 25.6 × 25.6 × 17.6 cm^3^, 256 × 256 imaging matrix in-plane, and 176 contagious sagittal slices at 1 mm thickness. 2D T2-weighted MRI was also acquired using a turbo-spin-echo sequence with the following parameters: TR = 6 s, TE = 73 ms, turbo factor = 9, echo spacing = 18.3 ms, field of view = 20.5 × 20.5 cm^2^, 256 × 256 imaging matrix, and 38 axial slices at 3 mm thickness. For functional MRI, blood-oxygen-level-dependent (BOLD) images were collected using a single-shot echo-planar-imaging (EPI) pulse sequence with the following parameters: TR = 2 s, TE = 26 ms, field of view = 20.5 × 20.5 cm^2^, 104 × 104 imaging matrix, and 28 contiguous 3 mm thick axial slices. Proton MR spectroscopy was performed in the visual cortex using the stimulated-echo-acquisition-mode (STEAM) pulse sequence by centering a single voxel mid-sagittally at the calcarine sulcus to cover both upper and lower visual field representations of both hemispheres with the following parameters: TR = 2 s, TE = 30 ms, mixing time (TM) = 10 ms, flip angle = 90°, voxel size = 20 × 25 × 30 mm^3^, water suppression bandwidth = 35 Hz, spectral bandwidth = 1200 Hz, 128 averages. The voxel of interest was positioned to exclude the lipid of the skull and the subcutaneous fat^38^,^39^. Diffusion tensor MR imaging was acquired covering the whole brain using spin-echo EPI diffusion-weighted sequences with the following parameters: 12 diffusion gradient directions at diffusion weighting factor (b) = 850 s/mm^2^ and one b = 0 s/mm^2^ (b_0_), TR = 5.2 s, TE = 80 ms, field of view = 20.5 × 20.5 cm^2^, 104 × 104 imaging matrix, and 38 axial slices at 3 mm thickness. We used anatomical landmarks based on T1-weighted MRI, T2-weighted MRI and the human brain atlas to ensure consistent localization of functional MRI, proton MR spectroscopy and diffusion tensor MRI measurements across subjects. The whole MRI experimental session lasted for about 1 hr for each subject.

### Visual presentation for functional MRI

For each eye, the activity within the visual cortex was assessed with a functional MRI experiment. Each scan was 5 min long consisting of 12 trials of alternating 12 s of rest and 12 s of visual stimulation. The visual stimuli were hemifield checkerboard patterns flashing at 8 Hz (Fig. 1a). The hemifield alternated between the upper and lower fields with each trial using the e-prime software (Psychology Software Tools, Inc., Sharpsburg, PA, USA). In order to ensure consistent head positioning and minimal motion artifacts, MRI-compatible visual occlusion spectacles (Translucent Technologies, Inc., Toronto, Ontario, Canada) were worn during experiments to present visual patterns to one eye at a time without the need to move the subjects in the scanner. We also used MRI-compatible correction spectacles during the whole experimental session to allow the subjects to maintain the best-corrected visual acuity of 20/40 or better in each eye for functional MRI tests.

### Data processing and statistical analysis

For functional MRI, image processing was performed using a combination of SPM8 subroutines ( www.fil.ion.ucl.ac.uk/spm/) and in-house software. Image volumes underwent slice timing correction to remove the timing errors between slices in each volume. Each volume in the time series was then realigned to the first volume using a 6 degree of freedom rigid body registration. The functional images were registered to the separately acquired T1-weighted MRI, and all these images were then normalized to the Montreal Neurological Institute (MNI) template using T1-weighted MRI as the reference image. Tissue probability maps were produced from the T1-weighted images using a unified segmentation algorithm^40^, which provided an estimated probability that each voxel belongs to a gray matter, white matter or cerebrospinal fluid (CSF) class. The images were then masked with a gray matter mask and smoothed with a Gaussian filter at full width half maximum = 5 mm.

We used a general linear model to calculate 4 activation maps for each combination of eye and visual field for each subject. The data for each eye were fit with a model that included the following predictors: an upper field boxcar function (equal to 1 when the subject was presented the upper field stimulus and equal to 0 otherwise) convolved with a hemodynamic response function, a lower field boxcar function (equal to 1 when the subject was presented the lower field stimulus) convolved with a hemodynamic response function, the temporal derivatives of these predictors, normalized motion parameters obtained during the realignment, the first and second temporal derivatives of the motion parameters, the average time courses in the white matter and CSF to control for physiological noise, and a constant. After solving for the predictor coefficients for each voxel, a map of BOLD % change was calculated for each combination of eye and field by taking the ratio of the coefficient of either predictor 1 for upper field map or predictor 2 for lower field map to the coefficient of the constant predictor. We then summarized visual cortex activation as the average BOLD % change over a number of regions of interest (ROIs). For each combination of eye and field, we created a group-wise activation map by performing a one-tailed, one-sample t-test and thresholding the map at a cluster level corrected p < 0.01 (cluster size ≥ 86 voxels). For each subject, we then calculated the average BOLD % change for each combination of eye and field by taking the average within a ROI that was the intersection of the group-wise activation map, and one of 3 Brodmann areas (BAs) defined by a standard atlas in MNI space. The BAs investigated include BA 17 (primary visual cortex), BA 18 (secondary visual cortex), and BA 19 (tertiary visual cortex).

For proton MR spectroscopy, peak integrals of visual brain metabolites [N-acetylaspartate (NAA), creatine (Cr), choline (Cho), glutamate/glutamine complex (Glx)] were determined using the syngo MR software (Siemens, Erlangen, Germany) after residual water filtering, apodization with a Hanning window, zero-filling from 1024 to 2048 spectral points, baseline correction, Fourier transformation, phase correction and curve fitting. The estimated brain metabolite levels were normalized to the Cr level to account for system stability and for quantitative comparisons with other modalities.

For diffusion tensor MRI, fractional anisotropy maps were obtained after image co-registration and transformation to align with the 1 × 1 × 1 mm^3^ MNI152 standard space, and were compared across groups using tract-based spatial statistics (TBSS)^41^ in FMRIB Software Library (FSL) ( http://www.fmrib.ox.ac.uk/fsl) and custom-written software. The averaged fractional anisotropy representing the overall white matter structural integrity was measured in the optic radiation for quantitative comparisons with other modalities.

We extracted the clinical OCT and visual field records to relate structural, metabolic and functional brain MRI findings with ophthalmic clinical findings using linear and broken-stick analyses based on data distribution. We used random intercept linear mixed-effects models to assess the structural, metabolic and functional brain relationships and eye-brain-behavior relationships among glaucoma and healthy subjects. We also compared visual field functions with OCT and functional MRI findings using a segmented regression model to determine the existence of a tipping point^13^. All analyses were conducted using *R* Language and Environment for Statistical Computing software^42^, and the *segmented* R package^43^,^44^. A P of 0.05 was the criterion for statistical significance.

## Additional Information

**How to cite this article**: Murphy, M. C. *et al.* Retinal Structures and Visual Cortex Activity are Impaired Prior to Clinical Vision Loss in Glaucoma. *Sci. Rep.* **6**, 31464; doi: 10.1038/srep31464 (2016).
